# Achievement of constitutive fluorescent pLEXSY-egfp *Leishmania braziliensis* and its application as an alternative method for drug screening in vitro

**DOI:** 10.1590/0074-02760160237

**Published:** 2017-02

**Authors:** Matheus Silva e Bastos, Luciana Ângelo de Souza, Thiago Souza Onofre, Abelardo Silva, Márcia Rogéria de Almeida, Gustavo Costa Bressan, Juliana Lopes Rangel Fietto

**Affiliations:** 1 Universidade Federal de Viçosa, Departamento de Bioquímica e Biologia Molecular, Viçosa, MG, Brasil; 2 Universidade Federal de Viçosa, Departamento de Veterinária, Viçosa, MG, Brasil

**Keywords:** Leishmania braziliensis, green fluorescent protein, drug screening

## Abstract

**BACKGROUND:**

Gene reporter-fluorescent cells have emerged as alternative method for drug screening.

**OBJECTIVE:**

Achievement of constitutive expression of fluorescent protein GFP by *Leishmania braziliensis* as alternative method for drug screening.

**METHODS:**

*L. braziliensis*-GFP was generated using *Leishmania tarentolae* pLEXSY-egfp for constitutive expression of GFP. Fluorescent cells were selected and subjected to standardisation tests of anti-promastigote and anti-intracellular amastigote assays.

**FINDINGS:**

Our results showed that *L. braziliensis*-GFP method is faster and more sensitive than Allamar Blue-resazurin.

**MAIN CONCLUSION:**

Transfected parasites maintained stable fluorescence after successive in vitro passages and pLEXSY system can be used to achieve non-*L. tarentolae* fluorescent cells.

Leishmaniasis is a vector-borne disease caused by more than 20 different species of protozoan parasites of the genus *Leishmania* (Herwaldt 1999). The World Health Organization has reported that approximately 1.3 million new cases of leishmaniasis occur each year and that 20,000 to 30,000 deaths occur during the same period as a consequence of the disease (WHO 2016). *Leishmania braziliensis* is the main species responsible for cutaneous leishmaniasis in the New World, and Brazil is one of the ten countries with the highest numbers of estimated cases (Alvar et al. 2012). *Leishmania* parasites have two main evolutionary forms: promastigotes, the flagellated form found in the sandfly vector, and amastigotes, the non-flagellated form present inside the mammalian host phagocytes. The control of leishmaniasis depends especially on chemotherapy because there is no effective and licensed human vaccine against the disease. In addition, there is a limited number of drugs that can be used in its treatment. Pentavalent antimony has long been used as the first line of treatment. Amphotericin B and other alternative drugs such as pentamidine, paromomycin and allopurinol are also employed. However, their use is limited due to different reasons, such as high cost, toxicity and parasite resistance, which has led to the search for new drugs for treating Leishmaniasis (Singh et al. 2012, Pace 2014).

The detection of cell viability has been used in the drug screening of antiparasitic and anticancer drugs (Bopp & Lettieri 2008).There are many colorimetric assays for screening of drugs against *Leishmania*, such as: lactate dehydrogenase assay (LDH), trypanothione reductase assay (TryR), ornithine decarboxylase assay (ODC), and those related to intracellular metabolising salts, like 3-(4,5-dimethyl thiazol-2-yl)-2,5-diphenyl tetrazolium bromide assay (MTT); 3-(4,5-dimethylthiazol-2-yl)-5-(3-carboxymethoxyphenyl)-2-(4-sulfophenyl)-2H-tetrazolium (MTS), and even those that employ resazurin (Ganguly et al. 2006 , Gould et al. 2008 , Rampersad 2012 , Kulshrestha 2013, van den Bogaart et al. 2014, Xu et al. 2015). Resazurin, known as Alamar Blue, are widely used in the screening of compounds with leishmanicidal potential because they are inexpensive and reproducible with good sensitivity and linearity (Gould et al. 2008 , Rampersad 2012). Resazurin is a sodium salt, and in its oxidised form is a blue dye. In the presence of cellular metabolic activity, it is reduced to resofurin that has a pink colour, which can be measured by a colorimetric or fluorometric reading (O’Brien et al. 2000). Despite the advantages of this methodology, some factors can influence its effectiveness, which must be carefully evaluated and standardised for each cell type. In this context, the temperature and time of incubation can influence the metabolism of resazurin as shown for *L. major* promastigotes that metabolise resazurin more slowly than blood forms of trypanosomes (Mikus & Steverding 2000 , Gould et al. 2008). In addition, during the drug screening, the compound itself may be able to reduce resazurin, leading to false-positive results. Moreover, using the resazurin method it is difficult to evaluate if the action of a drug led to cell death or simply led to cell growth arrest because killed cells may produce strong fluorescent signals, which is indicative of living cells (Gould et al. 2008). Finally, the screening of a large number of compounds by the resazurin method that assesses the action of these compounds at different times is laborious and requires a longer time. Thus, the search for faster alternative methodologies that are more direct and have higher performance has increased to reduce the limitations of the tests with resazurin (Rocha et al. 2013). Among these methods, the use of engineered cells expressing the green fluorescent protein (GFP) has emerged as a good alternative.

The green fluorescent protein is a reporter gene that has been extensively studied for protozoan parasites, and among its advantages are its low toxicity and easy quantification (Bolhassani et al. 2011). In *Leishmania*, the use of GFP has been shown for *L. major*, *L. donovani*, *L. infantum*, *L. mexicana*, and *L. amazonensis* (Rocha et al. 2013). A new heterologous expression system LEXSY using *L. tarentolae* was developed and allowed many different plasmids to be used for expression under the control of genome integration or the episomal way (Breitling et al. 2002).

In this work, we used one pLExsy construction to achieve *L. braziliensis* expressing GFP and then used it to evaluate its usefulness for the screening of leishmanicidal compounds against forms of the parasite including axenic promastigotes and intracellular amastigotes.

*Mammalian cells and parasite strain* - *L. braziliensis* MHOM/BR/75/M2904 and M2904-GFP promastigotes were maintained in Grace’s medium at 25ºC, and RAW 264.7 macrophages were kept in RPMI medium (RPMI-1640, Sigma, MO, USA) at 37ºC in a humid atmosphere containing 5% CO_2_. Both mediums were supplemented with 10% inactivated fetal calf serum (LGC Biotecnologia, SP, Brazil), L-glutamine (2 mM) (Serva Electrophoresis & Life Science Products, NY, USA) and penicillin (100 µg/ml) (USB Corporation, OH, USA).

*Transfection and growth* - *L. braziliensis* MHOM/BR/75/M2904 parasites were transfected with the pLExsy-EGFP-sat2 vector (Jena Bioscience, Thuringia, Germany) using 5 µg of SwaI digested vector following the manufacturer’s recommendations. The purification of digested plasmid was done using the PureLinkTM Quick Gel Extraction kit (Invitrogen, CA, USA). The selection of transfected cells was done using 100 µg/mL nourseothricin. Cells were incubated for 20 days at 25ºC. The recovery of the cells was daily checked by evaluating the GFP fluorescence under an EVOS fluorescence microscope (Life Technologies). After 20 days, cells were suspended in fresh supplemented Grace’s medium with nourseothricin. For the selection of single clones, we made a limiting dilution of transfected polyclonal cells previously selected with antibiotic. In a 96-well plate, 1 x 10^5^ cells were added to well A1, and then a serial dilution was made in all of the other remaining wells, which were considered clone isolated cells that were grown in wells where dilution generated cell numbers equal to 1 or 0. Following cell recovery and clonal selection, the cells were frozen. The polyclonal pool selected with antibiotic was subsequently used in drug trials. M2904 and M2904-GFP promastigotes of *L. braziliensis* were growth in three independent bottles with 10 mL of Grace’s medium each one. The bottles were keep at 25ºC. All six samples were counted using Neubauer chamber every day by eight days.

*Drug assay using L. braziliensis M2904 and M2904-GFP promastigotes -* Assays on promastigotes were made in 96-well plates and assessed after 24 and 48 h. Initially, a culture of 300 mL of *L. braziliensis* (wild type) was transferred to 50 mL sterile conical bottom tubes and centrifuged at 1,200 x *g* at 4ºC for 10 min. The obtained pellet was suspended in 5 mL of supplemented Grace’s medium. Subsequently, the cells were counted in a Neubauer chamber, and the volume was adjusted to achieve a concentration of 4 x 10^6^ *Leishmania*/well. Then, DMSO was added into control wells at a final concentration of 0.1%, and Amphotericin B diluted in DMSO 0.1% (Sigma-Aldrich) was added to the test wells at a final concentration of 3.125 µg/mL (3.4 µM). Subsequently, the plates were incubated at 25ºC for 23 and 47 h; then, 20 µL of resazurin (Sigma-Aldrich) was added, followed by incubation for 1 h. After 24 or 48 h, the plates were read on a microplate reader (Spectramax M5) at 570 nm and 600 nm at 1 h intervals for 3 h. For *L. braziliensis* GFP promastigotes, a culture of 30 mL was used. The cell culture was centrifuged and suspended as cited above. Subsequently, the cells were counted in the Neubauer chamber, and the volume was adjusted to 0.25 x 10^6^ cells/well. Then, DMSO and Amphotericin B were added at the same concentrations for *L. braziliensis* M2904 (wild type). Plates were then incubated at 25ºC for 24 and 48 h, and after these times, they were read on a microplate reader (Spectramax M5) with excitation at 490 nm and emission at 520 nm.

*Macrophage infection drug assay* - Infection was performed in 96-well plates. *Leishmania* and macrophages were cultured as described above. Initially, plates were assembled with 1 x 10^5^ macrophages/well and incubated at 37ºC for 24 h. Then, the infection of macrophages with *L. braziliensis* M2904-GFP promastigotes was performed, and a culture in a stationary phase (seven days) at a ratio of *Leishmania*:macrophage of 15:1 was obtained. Subsequently, plates were incubated at 37ºC for 24 h, and afterwards, they were washed 3x with RPMI. Then, supplemented RPMI was added, and the plates were incubated for 24 h at 37ºC. The metabolised medium was replaced by fresh medium, and DMSO at a final concentration of 0.1% was added to the control wells. In the test wells, 0.625 µg of Amphotericin B diluted in 0.1% DMSO was added. Internal controls were performed using infected macrophages and macrophages without any treatment. Subsequently, plates were incubated for 48 h and rinsed 3x with RPMI. Thereafter, we added 50 µL of lysis medium (RPMI unsupplemented + 0.05% SDS), and the plate was incubated at room temperature to achieve 90% lysis of the macrophages. Then, we added 150 µL of supplemented Grace’s medium, and the plate was sealed with parafilm followed by incubation at 25ºC for up to six days. *Leishmania* survival rates were estimated in relation to the controls and determined based on the fluorescence intensity after being read using a microplate reader (Spectramax M5) at 490 nm (excitation) and 520 nm (emission). In infection assays by the resazurin method using *L. braziliensis* M2904 (wild type), the assays were performed in the same manner cited above for *L. braziliensis* M2904-GFP. However, for up to the sixth day after incubation (which is sufficient time to have promastigotes forms), 20 µL of resazurin/well (Sigma-Aldrich) was added, and the plates were incubated for 1 h. After this, the plates were read on a microplate reader (Spectramax M5) at 570 nm and 600 nm at 1 h intervals for 3 h.

*IC*_*50*_ *and IC*_*90*_ *of amphotericin B* - Assay to determine the amphotericin B IC_50_ and IC_90_ (the concentration to inhibit parasite infection in 50% and 90%) of M2904 and M2904-GFP of *L. braziliensis* in in vitro infection were made using the same protocol described to the Macrophage infection drug assay. Time evaluated was 48 h. Amphotericin B was diluted from the stock-solution of 250 μg/mL and tested at the following concentrations: 1.25 μg/mL; 0.625 μg/mL; 0.3125 μg/mL; 0.15625 μg/mL; 0.078125 μg/mL; 0.0078125 μg/mL and 0.00078125 μg/mL. The positive control was the absence of amphotericin. The IC_50_ and IC_90_ were calculated using GraphPad Prism Version 7.0.

*L. braziliensis GFP achievement, selection and growth* - After achievement of transfected cells expressing the green fluorescent protein, four passages were made to ensure that all non-transfected cells were eliminated. Then, the clonal selection was performed by limiting dilution. We obtained nine isolated clones of *L. braziliensis* GFP, and one representative expanded clone is shown in Fig. 1A-B, where we can observe the GFP fluorescence of groups of promastigotes. In addition the insertion of GFP expression cassette do not influence the growth of promastigotes (Fig. 1C).

**Fig. 1:**
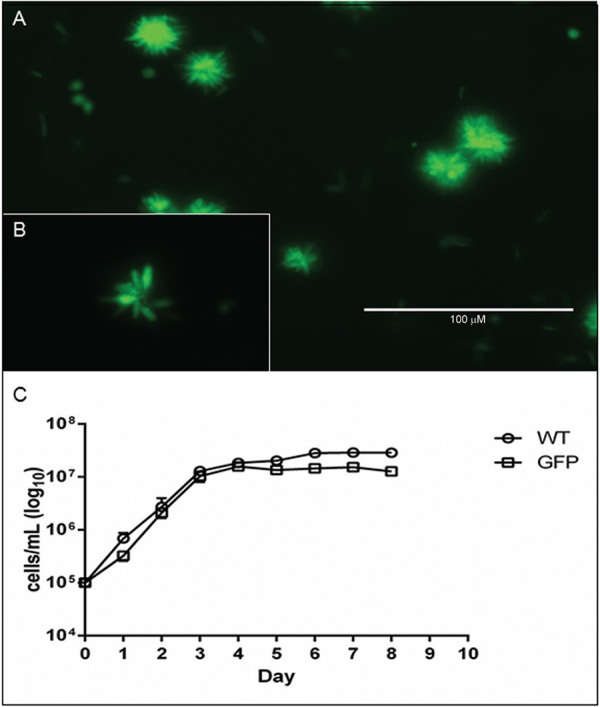
: *Leishmania braziliensis* GFP promastigotes and growth curve comparison with wild type. All promastigotes were cultivated in Grace’s medium supplemented with the selective antibiotic. (A) polyclonal promastigotes; (B) zoom of promastigote pool showing the classical form of promastigotes; and (C) growth curve comparison between wild type and GFP *L. braziliensis* promastigotes. Photos were taken using a green filter on an EVOS fluorescence microscope with magnifications of 60x (A). Scale bar: (A) 200 µM.

*Standardisation of the drug screening test using L. braziliensis GFP promastigotes* - In the standardisation of the drug screening assay using the transfected parasites, four different numbers of *L. braziliensis GFP* promastigotes were tested to start the assay: 1 x 10^6^, 0.5 x 10^6^, 0.25 x 10^6^ and 0.125 x10^6^ cells per well. These assays were performed to determine the best concentration of parasites to test drugs in comparison with non-transfected *L. braziliensis* promastigotes at a concentration of 4 x 10^6^ *Leishmania*/well evaluated by the resazurin method previously standardised. These results are shown in Table I and are evidence that the GFP fluorescence assay is more sensible than the resazurin assay. The GFP assay is able to detect the same level of action of the Amphotericin effect using an 8-16 times lower number of cells than the resazurin method (0.25 x 10^6^ cells for the GFP assay in contrast with 4 x 10^6^ cells for the resazurin assay after 24 h as well as 0.5 x 10^6^ cells compared to 4 x 10^6^ cells after 48 h, see Table I bolded numbers).

**TABLE I:** Comparison of the Leishmanicidal effect of Amphotericin B in promastigotes using the direct GFP fluorescence assay and indirect resazurin assay

Condition	GFP Fluorescent assay				Resazurin assay
# cell/well	1 x 10^6^	**0.5 x 10** ^**6**^	**0.25 x 10** ^**6**^	0.125 x 10^6^	4 x 10^6^
% live cells-24 h	53.44 ± 0.72	38.46 ± 0.87	**28.14** ± 1.27	26.58 ± 14.29	**27.84** ± 3.89
% live cells-48 h	50.11 ± 1.47	**33.09** ± 0.95	17.94 ± 1.20	11.19 ± 2.00	**31.40** ± 5.53

The assays were done using different number of *Leishmania* per well, as described in #cell/well. The data indicate that GFP fluorescence assay is more sensible than Resazurin because similar % of live cells were achieved using lower number of cells/well in GFP assay (highlighted in bold letters). The GFP is the assay using *L. braziliensis* M2904 expressing GFP; and Resazurin is the assay using non fluorescent *L. braziliensis* M2904 wild type strain. The data represent the Median and Standard Deviation of at least ten assays with internal quadruplicates for each of them. Percentage of live cells were estimated in comparison with data from the control (without Amphotericin).

*Drug assay for macrophage infection using L. braziliensis GFP* - In the infection assay using transfected parasites, *L. braziliensis* GFP promastigotes from the stationary phase containing infectious metacyclic forms were used to infect cultures of macrophages. After infection and the action of the control drug Amphotericin B, it was possible to observe the presence of fluorescent amastigotes within macrophages in the control sample as well as the absence or undetectable levels of intracellular forms in samples treated with Amphotericin B (Fig. 2). Similar results were observed after the measurement of the fluorescence of surviving parasites after the lyses of macrophages and proliferation of extra-cellular promastigotes derived from surviving intracellular amastigotes (Table II).

**Fig. 2:**
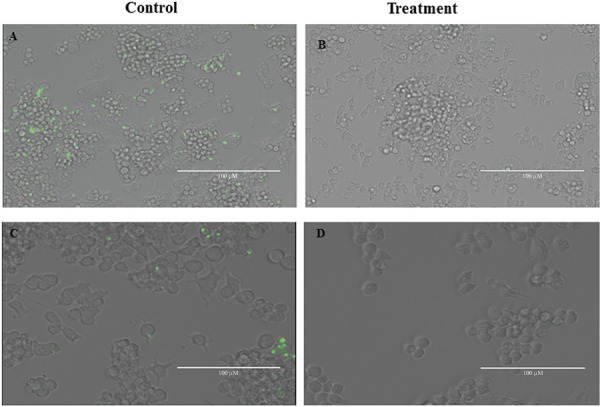
: visual evaluation of the macrophage infection assay using *Leishmania braziliensis* GFP. The images are the merge image from light microscopy image, showing the macrophages and immunofluorescence image from *L. braziliensis*-GFP intracellular amastigotes (green fluorescence). (A, C): visualisation of fluorescent intracellular amastigotes in the control sample (without Amphotericin B); (B, D): visualisation of the absence of fluorescent intracellular amastigotes in the treated sample (after treatment with Amphotericin B). Scale bar: 200 µM (A, B); 100 µM (C, D).

**TABLE II:** Amphotericin B Leishmanicidal effect in intracellular amastigotes using the direct GFP fluorescence assay

Treatment	% live cells	IC_50_	IC_90_
Amphotericin B	0 ± 2.8	0.34 µg/mL (0.37 µM)	3.06 µg/mL (3.33 µM)

The range of Amphotericin B concentration using in these assays were: 1.25 μg/mL; 0.625 μg/mL; 0.3125 μg/mL; 0.15625 μg/mL; 0.078125 μg/mL; 0.0078125 μg/mL and 0.00078125 μg/mL. The IC_50_ and IC_90_ were calculated using GraphPad Prism Version 7.0. The data represent the Median and Standard Deviation of at least three assays with internal quadruplicates for each of them. Percentage of live cells were estimated in comparison with data from the control (without Amphotericin B).

*Amphotericin B IC*_*50*_ *and IC*_*90*_ - We get dose-response curves of amphotericin B and calculated the IC_50_ and IC_90_ values using resazurin colorimetric assay and GFP-fluorescence assay of in vitro infection (promastigotes derived from intracellular amastigotes) after 48 h of amphotericin action. To the resazurin method IC_50_ was 0.77 µg/mL (0.84 µM) and IC_90_ was 6.93 µg/mL (7.56 µM). To the GFP-fluorescence method the IC_50_ was 0.34 µg/mL (0.37 µM) and IC_90_ was 3.06 µg/mL (3.33 µM) (Table II).

Development of new drugs for the treatment of leishmaniasis has required new methods for optimising the in vitro screening of compounds with leishmanicidal potential, and reporter genes such as GFP are promising tools for this purpose (Pulido et al. 2012). Herein, we report the generation of *L. braziliensis* expressing GFP, revealing that the fluorescent assay is more sensible and direct than the resazurin method. The compound screening assay in *Leishmania* promastigotes using the resazurin method requires mounting over a plaque to evaluate different times of action, as the resazurin is readily reduced by viable cells. Furthermore, long periods of resazurin incubation can make it toxic to cells and cause it to be carried to a second reduction stage, yielding hidroresofurin, a non-fluorescent and colorless product (O’Brien et al. 2000, Pace & Burg 2015), which makes it an infeasible test on a single plate for different time periods. However, when the screening is performed in promastigotes expressing GFP, the same plate can be used for all time analyses. In addition, the fluorescence measurement is predicted by the efficiency of compounds directly against the parasites and is not an indirect result as in the resazurin assay. The amphotericin B IC_50_ and IC_90_ determined to in vitro infection using resazurin method or GFP-fluorescence method emphasise the better usability of GFP in the optimisation of in vitro drug testing, since the value determined by GFP-fluorescence method was lower than that from resazurin method. This result could be explained by the needed of higher number of cells and by the presence of traces of lysed macrophages that could contribute to reduction of resazurin and may interfere in this method.

Thus, the GFP fluorescence assay was also adapted to be applied in an in vitro infection assay. The infection assay using the resazurin method has many problems, such as the difficultly of reproducibility, because the lysis of macrophages does not occur homogeneously, and the remaining cells not lysed on the plate can reduce resazurin and thus interfere with its reading. When the test is performed with parasite GFP, the macrophage lysis efficiency will not influence the fluorescence measurement. In addition, a control is used to subtract the intrinsic fluorescence of macrophages.

In conclusion, our results showed that the use of *L. braziliensis* GFP for screening drugs with potential against leishmaniasis is an affordable alternative, and it is more sensitive and faster in comparison to the resazurin method. In addition, the pLExsy plasmids can be used to produce stable *L. braziliensis* GFP parasites and may be useful for use in other non-*L. tarentolae* cells.
